# Taxonomical insights and ecology of sandfly (Diptera, Psychodidae) species in six provinces of Northern Vietnam

**DOI:** 10.1051/parasite/2021080

**Published:** 2021-12-17

**Authors:** Sinh Nam Vu, Hai Son Tran, Vu Phong Tran, Cong Tu Tran, Nhu Duong Tran, Duc Anh Dang, Thi Yen Nguyen, Thi Lieu Vu, Khanh Phuong Ngo, Viet Hoang Nguyen, Ngọc Anh Hoàng, Cécile Cassan, Jorian Prudhomme, Jérôme Depaquit, Nil Rahola, Anne-Laure Bañuls

**Affiliations:** 1 National Institute of Hygiene and Epidemiology 1 Yec-Xanh Street, Hai Ba Trung District 100000 Hanoi Vietnam; 2 MIVEGEC, UMR Univ Montpellier-IRD-CNRS, Centre IRD Montpellier – 911 Avenue Agropolis BP64501 34394 Montpellier Cedex 05 France; 3 EA7510 ESCAPE, USC ANSES “VECPAR”, UFR Pharmacie, Université de Reims Champagne-Ardenne 51096 Reims France

**Keywords:** Sandfly, Spatial distribution, *Sergentomyia*, *Phlebotomus*, Leishmaniasis risk, Northern Vietnam

## Abstract

We studied sandfly (Diptera: Psychodidae) populations in six provinces of Vietnam. This work explores the diversity of sandfly species according to the province, as well as environment, and updated information on public health since leishmaniasis cases were reported in two provinces. Sandflies were collected using 428 CDC light traps from May 30 to October 13, 2016 and identified based on the morphology of the cibarium, pharynx and/or male genitalia or female spermathecae. A total of 2585 sandflies belonging to five genera and 13 identified species were collected. The main species were: the *Sergentomyia barraudi* group (12.53%), *Se. sylvatica* (9.63%) and *Phlebotomus stantoni* (3.95%). In all, 294 *Sergentomyia* specimens classified as *Se.* sp2 and *Se.* sp3 and a heterogeneous group, herein called *Se.* und_sp*.*, showed unknown morphological characteristics requiring further studies. We provide detailed comments about morphological description and taxonomical identification in order to help standardization of sandfly classification in Southeast Asia. We observed differentiation according to the provinces in terms of density and species richness, with Lang Son having the highest density and Ninh Binh having the highest species richness. The majority of specimens were collected in rock caves and outdoors, suggesting mainly cavernicolous and exophilic characters of sandfly species in Northern Vietnam. However, specimens were also collected in intra- and peri-domiciliary sites. It is worth noting that *Ph. stantoni* was the main species found in dog sheds and indoors, and in particular in a leishmaniasis patient’s house.

## Abstract (fr)

Nous avons étudié les populations de phlébotomes (Diptera : Psychodidae) dans six provinces du Vietnam. Ce travail explore la diversité des espèces de phlébotomes selon les provinces, les environnements et les informations mises à jour sur la santé publique depuis que des cas de leishmanioses ont été signalés dans deux provinces. Des phlébotomes ont été collectés à l’aide de 428 pièges lumineux CDC du 30 mai au 13 octobre 2016 et identifiés sur la base de la morphologie du cibarium, du pharynx et/ou des organes génitaux des mâles ou des spermathèques des femelles. Au total, 2585 phlébotomes, appartenant à cinq genres et 13 espèces identifiées, ont été collectés. Les principales espèces étaient : le groupe *Sergentomyia barraudi* (12,53 %), *Se. sylvatica* (9,63 %) et *Phlebotomus stantoni* (3,95 %). 294 spécimens de *Sergentomyia* classés comme *Se.* sp2 et *Se.* sp3 et un groupe hétérogène ici appelé *Se.* und_sp. ont montré des caractéristiques morphologiques inconnues nécessitant des études complémentaires. Nous fournissons des commentaires détaillés sur la description morphologique et l’identification taxonomique afin d’aider à la normalisation de la classification des phlébotomes en Asie du Sud-Est. Nous avons observé une différenciation selon les provinces en termes de densité et de richesse en espèces, avec Lang Son ayant la plus forte densité et Ninh Binh ayant la plus grande richesse en espèces. La majorité des spécimens ont été collectés dans des grottes rocheuses et à l’extérieur, suggérant principalement des caractères cavernicoles et exophiles des espèces de phlébotomes du nord du Vietnam. Cependant, des spécimens ont également été collectés dans des sites intra et péri-domiciliaires. Il convient de noter que *Ph. stantoni* était la principale espèce trouvée dans les abris pour chiens et à l’intérieur, notamment dans la maison d’un patient atteint de leishmaniose.

## Introduction

Sandflies are known to be vectors transmitting leishmaniases and other pathogens to humans and animals. These small insects belong to the Diptera order, Psychodidae family [12, 49] and the Phlebotominae subfamily [14]. In 2015, Hotez et al. emphasized the need to increase surveillance activities on neglected tropical diseases, among which leishmaniases, in the Association of Southeast Asian Nations (ASEAN) region, where the extent of the burden is far from known [11].

Leishmaniasis is a public health problem in many Asian countries, such as in Western and Central China [23, 24] where more than 40 species of sandflies have been identified. Five of them were identified as vectors of visceral leishmaniasis, i.e. *Phlebotomus* (*Adlerius*) *chinensis*, *Ph*. (*Adl*.) *longiductus*, *Ph*. (*Adl*.) *sichuanensis*, *Ph*. (*Paraphlebotomus*) *alexandri*, and *Ph*. (*Larroussius*) *smirnovi* [8, 25, 50]. In Southeast Asia, the first visceral leishmaniasis case was reported in Thailand in 1996 [47] and since then, 16 symptomatic cases have been notified [18]. An entomological survey revealed that the *Se*. (*Neophlebotomus*) *gemmea* species was the most predominant species in which *Leishmania* DNA was identified [17]. Nevertheless, Depaquit et al. recently suggested the need to reconsider the identification of this species, underlining that much remains to be done on the classification and determination of *Leishmania* vector species in Southeast Asia [7].

In Vietnam, a few entomological surveys conducted by Raynal and Gaschen 1935, Parrot and Clastrier 1952, 1962, Lewis 1978, Lewis 1982, 1987 [20–22, 28, 33, 35] described 11 sandfly species in 18 locations of Northern Vietnam: *Ph.* (*Anaphlebotomus*) *stantoni*, *Ph.* (*Euphlebotomus*) *argentipes*, *Se.* (*Parrotomyia*) *barraudi*, *Se.* (*Par.*) *brevicaulis*, *Se.* (*Neo.*) *hivernus*, *Se.* (*Neo.*) *iyengari*, *Se.* (*Neo.*) *perturbans*, *Se.* (*Neo.*) *sylvatica*, *Se.* (*Neo.*) *tonkinensis*, *Se.* (*Ser.*) *bailyi*, and *Se.* (*Ser.*) *morini*. These species have also been included in the list of sandflies described in the Old World by Seccombe, Ready and Huddleston in 1993 [38]. Among them, *Ph. argentipes* was described as a potential vector of *Leishmania* in India [16]. In 2019, our team published a first paper on the diversity and ecology of sandflies in Quang Ninh Province, Vietnam [49]. In this paper, four genera were collected and 10 sandfly species were identified: *Se. sylvatica*, *Se. barraudi*, *Se. hivernus*, *Se. bailyi*, *Ph. mascomai*, *Ph. stantoni*, *Ph. yunshengensis*, *Ph. betisi*, *Chinius junlianensis*, and *Idiophlebotomus longiforceps*. In this province, three visceral leishmaniasis cases were reported in 2001 [10]. However, a series of surveys in the same province on humans, dogs, rodents and sandflies did not detect the presence of *Leishmania* parasites in this locality [4]. Another case of visceral leishmaniasis was reported in the Quang Binh province in 2018, Central Vietnam [48], 700 km south from the first three cases. The question of *Leishmania* transmission is therefore still open in Vietnam and the data on sandflies remain incomplete considering the high biodiversity of the ecosystems in the country. This paper provides information on the taxonomy and geographical and environmental distribution of sandflies in 6 northern provinces of Vietnam, including data on Quang Ninh province published in 2019 [49].

## Material and methods

### Study sites

**Figure 1.**
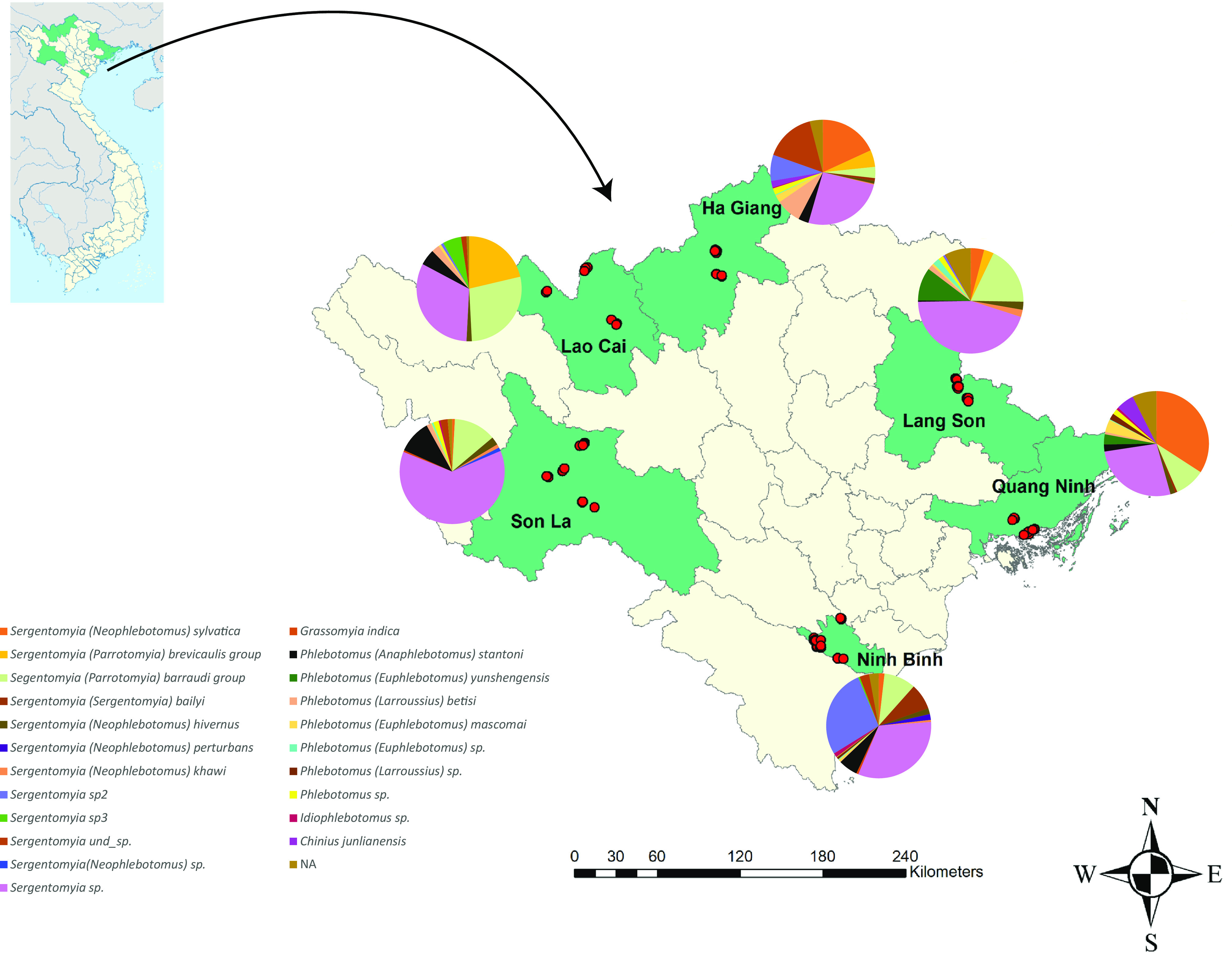
Collection sites and sandfly species composition in 6 provinces of Northern Vietnam.

The 6 provinces: Ninh Binh, Son La, Lang Son, Ha Giang, Lao Cai and Quang Ninh are located in the north of Vietnam, bordered by China (Lang Son, Lao Cai, Ha Giang and Quang Ninh) and by Lao PDR (Son La). These provinces were selected to represent the geographical and environmental diversity of Northern Vietnam (see Fig. 1). For each site, all details such as coordinates, environments, animals and the descriptions of trap locations, were recorded (see Table S1). All the data collected in the first study in Quang Ninh province were included in this paper in order to give an overview of the spatial and environmental distribution of sandflies in Northern Vietnam ([49] and Table S1).

### Sampling

CDC miniature light traps (LT, John W. Hock Co. FL, U.S.A.) were used to collect sandflies from May 30 to October 13, 2016 at 116 sites distributed in the 6 provinces (Fig. 1). At each sampling site, 2–9 LTs were installed depending on the site environment (inside and/or outside houses, animal barns, caves, crevices in walls) and were operated overnight from 6:00 pm to 09:00 am the next day. In total, 428 LTs were set up at the 116 sites (see Table S1).

After each night of trapping, captured specimens were transferred individually into 1.5 mL Eppendorf tubes containing 90% ethanol and labeled. Prior to mounting, the sandfly heads and genitalia were removed. Bodies were stored separately for future diagnostic analysis. The heads and genitalia were mounted in Euparal after different successive baths: 2 h in 10% potassium hydroxide, 2 h in distilled water, 10 h in a Marc-André solution [1], 10 h in distilled water, 20 min in 70% ethanol, 20 min in 90% ethanol, 20 min in 100% ethanol, and 10 h in a clove oil solution. Specimen identification was individually verified based on the morphology of the cibarium, pharynx and/or the male genitalia or female spermathecae, as described by Abonnenc (1972), Johnson (1991) and Lewis (1982) [1, 13, 21].

### Data analysis

Different parameters were calculated for characterizing sandfly populations at the different sites: density of collected sandflies (number of specimens per trap and per night); relative abundance (number of specimens of species/total number of specimens × 100); species richness (number of species in a given area). A Kruskal–Wallis test was used to compare the distribution of sandflies by province and according to the environment.

## Results

### Species and genus composition, density and abundance

A total of 2585 sandfly specimens, including 1511 males (58.5%) and 1049 females (40.5%) specimens, corresponding to a ratio of 1.44, were collected from the 116 sites. We could not define the gender for 25 specimens because of damaged organs. The overall sandfly density for the 428 traps and the 23 nights of trapping was 0.26.

In total, 2458 specimens were identified to the genus level and among these, 1090 were identified to the species level. One hundred and twenty-seven specimens (4.91%) could not be identified because of missing or damaged parts. The 1157 specimens characterized only to the genus or subgenus level (defined as sp. and und_sp.), as expected, were mainly *Sergentomyia* males (80.21%) (Tables 1 and S1).

**Table 1.** Number of specimens per taxon, sex ratio, density and relative abundance according to the taxa.

*Species*	Number	Female/Male ^(*n)^	Density	Relative abundance
*Sergentomyia* (*Neophlebotomus*) *sylvatica*	249	87/161^(*1)^	0.0253	9.632
*Sergentomyia* (*Parrotomyia*) *brevicaulis* group	66	49/17	0.0067	2.553
*Sergentomyia* (*Parrotomyia*) *barraudi* group	324	303/21	0.0329	12.534
*Sergentomyia* (*Sergentomyia*) *bailyi*	55	44/11	0.0056	2.128
*Sergentomyia* (*Neophlebotomus*) *hivernus*	49	46/3	0.0050	1.896
*Sergentomyia* (*Neophlebotomus*) *perturbans*	11	5/6	0.0011	0.426
*Sergentomyia* (*Neophlebotomus*) *khawi*	25	7/18	0.0025	0.967
*Sergentomyia* sp2	201	140/58^(*3)^	0.0204	7.776
*Sergentomyia* sp3	10	8/2	0.0010	0.387
*Sergentomyia* und_sp	83	65/18	0.0084	3.211
*Sergentomyia* (*Neophlebotomus*) sp*.*	4	4/0	0.0004	0.155
*Sergentomyia* sp*.*	990	50/928^(*12)^	0.1006	38.298
*Grassomyia indica*	6	1/5	0.0006	0.232
*Phlebotomus* (*Anaphlebotomus*) *stantoni*	102	46/55^(*1)^	0.0104	3.946
*Phlebotomus* (*Euphlebotomus*) *yunshengensis*	87	28/59	0.0088	3.366
*Phlebotomus* (*Larroussius*) *betisi*	50	3/47	0.0051	1.934
*Phlebotomus* (*Euphlebotomus*) *mascomai*	35	17/18	0.0036	1.354
*Phlebotomus* (*Euphlebotomus*) sp*.*	21	5/16	0.0021	0.812
*Phlebotomus* (*Larroussius*) sp*.*	12	4/8	0.0012	0.464
*Phlebotomus* sp*.*	33	25/8	0.0034	1.277
*Idiophlebotomus* sp*.*	14	6/8	0.0014	0.542
*Chinius junlianensis*	31	27/4	0.0031	1.199
NA	127	79/40^(*8)^	0.0129	4.913
Total	2585	1049/1511^(*25)^	0.2626	100

*^n^Number of specimens for which the gender could not be determined, NA: Not Available.

**Figure 2.**
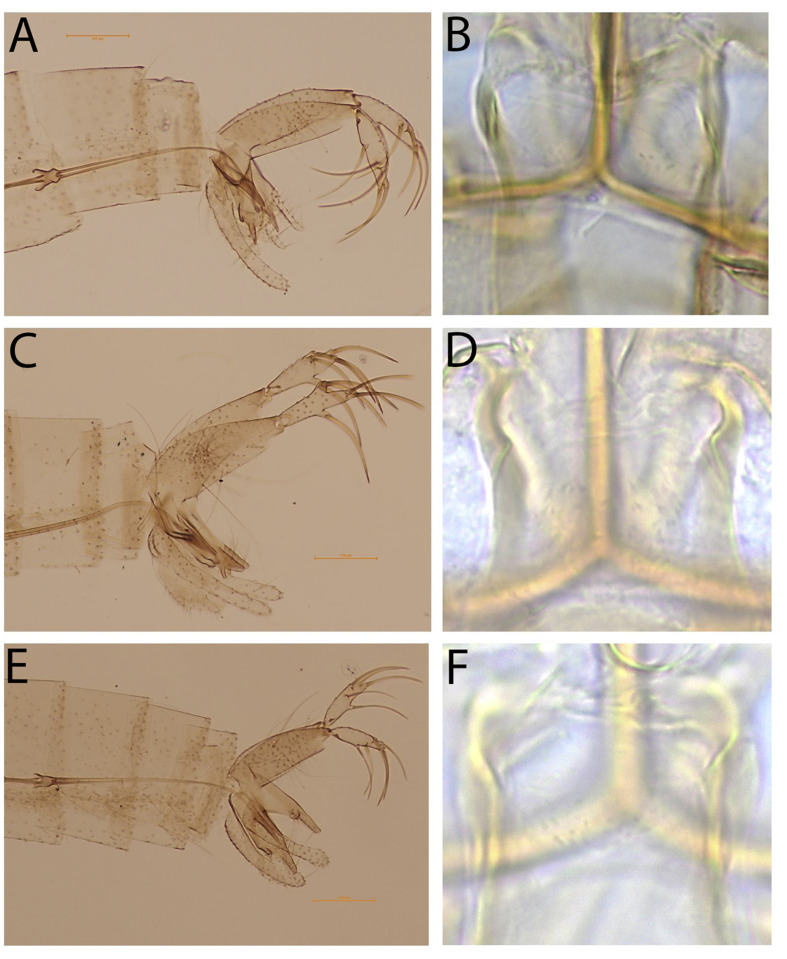
Classical characteristics of *Phlebotomus mascomai*, *Ph. yunshengensis* and *Ph. betisi* males. A and B: Genitalia and cibarium of *Ph. mascomai* male, respectively; C and D: Genitalia and cibarium of *Ph. yunshengensis* male, respectively; E and F: Genitalia and cibarium of *Ph. betisi* male, respectively*.*

The main genus found was *Sergentomyia* (*n* = 2067, 79.96%), followed by *Phlebotomus* (*n* = 340, 13.15%), *Chinius* (*n* = 31, 1.2%), *Idiophlebotomus* (*n* = 14, 0.54%), and *Grassomyia* (*n* = 6, 0.23%) (Table 1). In total, thirteen species could be identified, among which 7 *Sergentomyia* species which consisted of 779 specimens (Tables 1 and S1). The *Se. barraudi* group and *Se. sylvatica* were the predominant species with 324 and 249 specimens, respectively. We used the *Se. barraudi* group as a designation since we observed morphological variabilities in this taxon (data not shown). Five other species accounted for 206 specimens including *Se. bailyi* (*n* = 55), *Se. hivernus* (*n* = 49), *Se. brevicaulis* (*n* = 66), *Se. khawi* (*n* = 25), and *Se. perturbans* (*n* = 11). Four species accounting for another 274 specimens belonged to the *Phlebotomus* genus: *Ph. stantoni* (*n* = 102), *Ph. yunshengensis* (*n* = 87), *Ph. betisi* (*n* = 50), *Ph. mascomai* (*n* = 35) and unknown *Phlebotomus* species (*n* = 66). Figure 2 illustrates the classical characteristics of *Ph. mascomai*, *Ph. yunshengensis* and *Ph. betisi*. Additionally, *Ph. stantoni* and *Se. sylvatica* were previously illustrated in Vu et al. [49]. Two other genera, consisting of 37 specimens, were represented by only one species each, *Ch. junlianensis* and *Gr. indica* (Table 1)*.* The 14 specimens of the last genus, *Idiophlebotomus*, could not be validated with certainty to the species level and were named *Idiophlebotomus* sp. (see Table S1 and below). Moreover, 211 specimens (8.16%), classified as *Se.* sp2 and *Se.* sp3, exhibited morphological characteristics difficult to attribute to existing species (Tables 1 and S1). It is worth noting that the specimens described as Sp1 in Vu et al. [49], after detailed study, are ultimately thought to be females of *Ph. yunshengensis* (see discussion for more details).

### Sandfly diversity between provinces

The number of specimens was different according to the province. In all, 416 specimens were collected in Quang Ninh province using 68 light traps for 4 nights, 612 were collected in Ninh Binh province with 85 light traps for 4 nights, 728 specimens were collected in Lang Son province using 76 light traps for 4 nights, 122 specimens were collected in Lao Cai province using 74 light traps for 4 nights, 347 specimens were collected in Ha Giang province using 53 light traps for 3 nights, and 360 specimens were collected in Son La province using 72 light traps for 4 nights (see Tables 2 and S1).

**Table 2:** Number of specimens per taxon, relative abundance, density and species richness per province

*Species*	Quảng Ninh	Ninh Bình	Lạng Sơn	Lào Cai	Hà Giang	Sơn La
*Sergentomyia* (*Neophlebotomus*) *sylvatica*	142	11	30		63	3
*Sergentomyia* (*Parrotomyia*) *brevicaulis* group			22	26	18	
*Sergentomyia* (*Parrotomyia*) *barraudi* group	39	60	132	34	12	47
*Sergentomyia* (*Sergentomyia*) *bailyi*	3	49			3	
*Sergentomyia* (*Neophlebotomus*) *hivernus*	6	11	18	2	3	9
*Sergentomyia* (*Neophlebotomus*) *perturbans*		11				
*Sergentomyia* (*Neophlebotomus*) *khawi*		4	16		1	4
*Sergentomyia* sp2		168	4	1	28	
*Sergentomyia* sp3		3		7		
*Sergentomyia* und_sp		18	1	2	54	8
*Sergentomyia* (*Neophlebotomus*) sp*.*						4
*Sergentomyia* sp*.*	112	199	326	39	89	225
*Grassomyia indica*		4				2
*Phlebotomus* (*Anaphlebotomus*) *stantoni*	9	36	3	6	11	37
*Phlebotomus* (*Euphlebotomus*) *yunshengensis*	13		74			
*Phlebotomus* (*Larroussius*) *betisi*	3	1	11	3	27	5
*Phlebotomus* (*Euphlebotomus*) *mascomai*	16	4	5	1	8	1
*Phlebotomus* (*Euphlebotomus*) sp*.*	1	2	15		1	2
*Phlebotomus* (*Larroussius*) sp*.*	8	4				
*Phlebotomus* sp*.*	7	1	12		7	6
*Idiophlebotomus* sp.	3	8			1	2
*Chinius junlianensis*	23		1		7	
NA	31	18	58	1	14	5
Total	416	612	728	122	347	360
Relative abundance	16.09	23.68	28.16	4.72	13.42	13.93
Density	1.53	1.80	2.39	0.41	2.18	1.25
Species Richness*	9	12	11	8	11	8

* All the identified species were used for the calculation, including *Se*. sp2 and *Se*. sp3.

The *Sergentomyia* genus was the most predominant genus in the 6 provinces followed by the *Phlebotomus* genus. It is worth noting that distribution according to the genus between provinces was not significantly different. Furthermore, the distribution of specimens according to species identification, including *Se*. sp2 et *Se*. sp3, was significantly different between provinces (*p*-value = 0.002, *α* = 0.05 see Fig. 1 and Table 2). As examples, *Se. bailyi* and *Se.* sp2 were mainly detected in Ninh Binh province, *Se. barraudi* group in Lang Son and *Se. sylvatica* in Quang Ninh. The highest species richness (SR = 12) was observed in Ninh Binh, whereas the highest relative abundance and density were detected in Lang Son (RA = 28.16, *D* = 2.39, respectively).

### Collection sites and habitat preferences

At each of the 428 sites, light traps were installed in different environments, indoors, outdoors, in animal sheds or caves. Depending on the station, the number of light traps varied from 2 to 9.

The data showed that in the 6 provinces, sandflies were mainly collected in caves (*n* = 1431, relative abundance = 55.36 and *D*_cave_ = 0.79, Table 3) with the highest species richness (SR_cave_ = 15, including *Se*. sp2 et *Se*. sp3). Regarding relative abundance, we also collected numerous sandflies outdoors (in gardens), with 936 specimens corresponding to a relative abundance of 36.21 and a species richness of 15 (including *Se.* sp2 and *Se.* sp3). Nevertheless, sandfly density was higher in dog sheds (*D*_dogs_ = 0.36 vs. *D*_outdoor_ = 0.23, Table 3). The density of sandflies indoors was weak and similar to the density in chicken/bird/duck sheds and lower than the density in buffalo/cow/goat sheds (*D*_indoor_ = 0.08; *D*_chicken/bird/duck_ = 0.10; *D*_buffalo/cow/goat_ = 0.12; see Table 3). This distribution according to the environment was similar (no significant differences) between the 6 provinces.

**Table 3:** Number of specimens per taxon, relative abundance, mean value (number of sandflies/number of CDC traps), density and species richness according to different environments.

*Species*	Buffalo/cow/goat shed	Cave	Chicken/bird/duck shed	Dog shed	Indoor area	Outdoor area (garden)	Pig shed
*Sergentomyia* (*Neophlebotomus*) *sylvatica*	1	141	14	1		92	
*Sergentomyia* (*Parrotomyia*) *brevicaulis* group		45				21	
*Sergentomyia* (*Parrotomyia*) *barraudi* group	4	172	4		1	138	5
*Sergentomyia* (*Sergentomyia*) *bailyi*	15	10	5		3	17	5
*Sergentomyia* (*Neophlebotomus*) *hivernus*	3	10	5	1		28	2
*Sergentomyia* (*Neophlebotomus*) *perturbans*		6			1	4	
*Sergentomyia* (*Neophlebotomus*) *khawi*	3	16			1	5	
*Sergentomyia* sp2	5	135	2		3	55	1
*Sergentomyia* sp3		9				1	
*Sergentomyia* und_sp.		20	1		2	60	
*Sergentomyia* (*Neophlebotomus*) sp.		4					
*Sergentomyia* sp.	12	566	12	2	6	378	14
*Grassomyia indica*		4				2	
*Phlebotomus* (*Anaphlebotomus*) *stantoni*	11	32	8	7	11	24	9
*Phlebotomus* (*Euphlebotomus*) *yunshengensis*		80		1		6	
*Phlebotomus* (*Larroussius*) *betisi*		19	1			30	
*Phlebotomus* (*Euphlebotomus*) *mascomai*	2	24		1		8	
*Phlebotomus* (*Euphlebotomus*) sp.		14	2			4	1
*Phlebotomus* (*Larroussius*) sp.		10	1	1			
*Phlebotomus* sp.	3	15	2	1		11	1
*Idiophlebotomus* sp.		7				7	
*Chinius junlianensis*	1	24				6	
NA	5	68	9	1	2	39	3
Total	65	1431	66	16	30	936	41
Relative abundance	2.51	55.36	2.55	0.62	1.16	36.21	1.59
Density	0.12	0.79	0.10	0.36	0.08	0.23	0.07
Mean (SF nb/CDC nb)	1.44	15.66	1.57	1.78	1.07	5.29	1.14
Species Richness*	9	15	7	5	6	15	5

* All the identified species were used for the calculation, including *Se*. sp2 and *Se*. sp3.

In caves, all the genera and species were found (see Tables 3 and S1). Nevertheless, the *Sergentomyia* genus was the most represented, followed by the *Phlebotomus* genus. In dog sheds and indoors, the most represented sandfly was *Ph. stantoni* (*n* = 7 out of 16 and *n* = 11 out of 30, respectively) (Tables 3 and S1). As described in Vu et al. [49], two *Ph. stantoni* specimens were found in the house of one of the patients infected by leishmaniasis in 2001 [10]. It is worth noting that *Se.* sp2, *Se.* sp3 and the *Se.* und_sp*.* were mainly detected in caves and outdoors. The statistical analyses showed that the distribution of species was globally different according to the environment (*p*-value < 0.01, *α* = 0.01).

## Discussion

Only a few studies on sandflies have been carried out in Vietnam since three autochthonous cases of visceral leishmaniasis were reported in Quang Ninh province in 2001 [10]. At that time, a survey on humans, dogs, rodents and sandflies was carried out in Quang Ninh province that confirmed the presence of sandflies, but not the presence of parasites [4]. Since then, no further information has been gathered regarding the diversity, ecology and medical significance of these insects. We recently published data obtained in Quang Ninh province [49]. This study showed that sandflies are abundant in Quang Ninh province, that some species are found close to domestic animals and inhabitants, and that we still have a lot to learn about sandflies in Vietnam. To complete the picture on the ecology, distribution and diversity of sandflies in Northern Vietnam, we extended the study to five other northern provinces, Ninh Binh, Son La, Lang Son, Ha Giang, and Lao Cai. The data obtained in the six provinces were combined in this paper to improve knowledge on the taxonomy and the ecology of sandflies, but also to explore the risk of *Leishmania* transmission.

### Taxonomy

Until now, 11 species have been reported in Vietnam (see above). In our study, by combining the data obtained in the six provinces, 13 species could be identified of which seven were already described in Vietnam (*Se. barraudi* group, *Se*. *brevicaulis*, *Se. hivernus*, *Se. perturbans*, *Se. sylvatica*, *Ph. Stantoni*, and *Ph. yunshengensis*). Six species or genera were not previously reported (*Se. bailyi*, *Se. khawi*, *Ph. mascomai*, *Ph. betisi*, *Ch. junlianensis*, *Gr. indica*, and *Idiophlebotomus* sp.), and two taxa could belong to new *Sergentomyia* species, *Se.* sp2 and *Se.* sp3. We created a last group called “*Se*. und_sp*.*” which is composed of specimens with heterogeneous characters (see below). These specimens will be further described by molecular characterization.

In this study, the main species in northern Vietnam which are characterized are *Se. barraudi* group (*n* = 324), *Se. sylvatica* (*n* = 249) and *Ph. stantoni* (*n* = 102). A total of 294 specimens showing unknown morphological characters were classified in the groups named *Se*. sp2, *Se*. sp3 and *Se*. und_sp. The main one is *Se.* sp2 with 201 specimens and *Se.* und_sp. with 83 specimens. These specimens need to be further studied. Molecular methods and detailed morphological characterization will be carried out to define their taxonomic status. As described in our previous paper, finding new sandfly taxa was to be expected since, so far, only few studies have been carried out in Vietnam but also in Southeast Asia [49]. Nevertheless, based on the small number of publications, the high diversity of sandflies and the lack of data show that it is essential to entirely revisit the sandfly classification in Southeast Asia and at a larger scale in Asia for consistency of identifications. This will help determine identification keys for Asian sandfly species as underlined in the research of Phumee et al. in Southern Thailand [30]. The need for standardized species criteria in this region is highlighted by recent work that shows the misidentification of *Se. gemmea* species in Thailand [7].

We added below some comments about morphological description and taxonomical identification.

#### Comments about the *Phlebotomus* species

*Phlebotomus stantoni* is a common species easy to identify definitively.

The identification of *Ph. mascomai* seems logical in terms of biogeography because Vietnam is not far from Thailand, the country where this species was described for the first time. Importantly, *Ph. mascomai* is a species closely related to *Ph. argentipes*. Misidentifications are possible. *Phebotomus mascomai* differs from *Ph. argentipes* by the antennal formula in both genders; by a greater number of rings of the spermathecae form the female; by the position of the spines on the gonostyle, the absence of lateral spine of the paramere, and by the length of the aedeagal ducts [26].

The identification of *Ph. betisi* is easy since it is the only species belonging to the subgenus *Larroussius* recorded in Southeast Asia. In addition, some characters differ from the other members of the subgenus i.e. the neck of the spermathecae or the position of the spines in the gonostyle [15].

We caught 59 males of *Ph. yunshengensis* in the present paper and 28 sympatric females exhibiting characters never previously observed in described species. Consequently, according to their sympatry with the *Ph. yunshengensis* males, we tentatively describe the female as being that of *Ph. yunshengensis*. The description and anatomical nomenclature are in agreement with the latest guidelines [9].

The counts and measurements provided below are those of the specimen labelled M1-10-58.

Information about thorax, legs and wings could not be added because they were kept for molecular analyses.

#### Head

Occiput with two narrow lines of well individualized setae.

Clypeus 112.5 μm long, 68.7 μm wide with 20 setae randomly distributed.

Eyes 208 μm long, 83 μm wide with about 90–100 facets.

Incomplete interantennal suture.

Complete interocular suture but not reaching the interantennal one.

**Figure 3.**
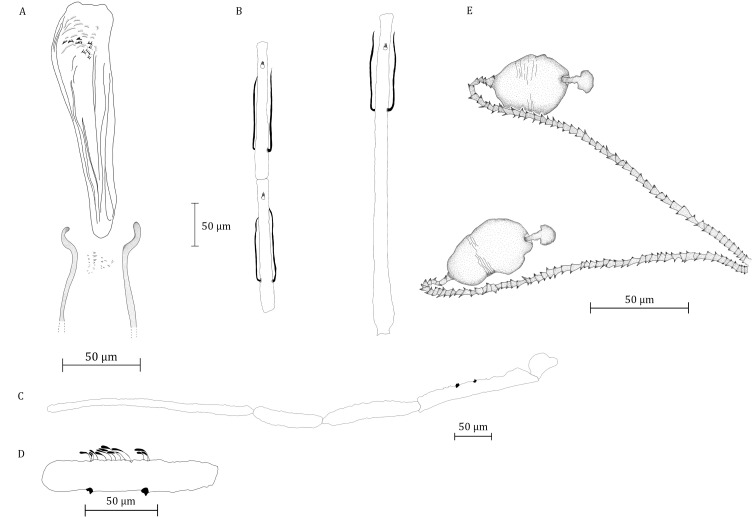
*Ph. yunshengensis* female. A: pharynx and cibarium; B: flagellomeres 1, 2, and 3; C: palp; D: detail of 3rd palpal article; E: spermathecae.

Cibarium (Fig. 3A) armed with a group of about 25 discrete teeth. Absence of pigment patch.

Pharyngeal armature (Fig. 3A) made with small dots-like teeth at the back and some short anterior grouped triangular teeth.

Flagellomeres (Fig. 3B) f1 = 369 μm, f2 = 154 μm, f3 = 158.3 μm. Flagellomere 1 longer than f2 + f3. Ascoidal formula: 2/f1–f7 with long ascoids, not reaching the next article, flagellomeres broken after f9.

One distal papilla on f1, f2 and f3. Absence of simple setae on flagellomeres f1, f2 and f3. One simple seta on flagellomeres f4, to f7.

Palpi (Figs. 3C, 3D) p1 = 33 μm, p2 = 164 μm, p3 = 120 μm, p4 = 97 μm, p5 = 283 μm. Palpal formula: 1, 4, 3, 2, 5. Presence of two groups of about 10 and 3 club-like Newstead’s sensillae in the middle of the third palpal segment. No newstead’s sensilla on other palpal segment. Presence of one spiniform setae on p3; 4 on p4 and 8 on p5.

Labrum-Epipharynx 223 μm long. f1/*E* = 1.65.

Labium: labial furca closed.

#### Abdomen

VIII tergite: presence of 30 setae each side.

Tergite IX without any protuberance.

#### Genital apparatus (Fig. 3E)

Thin-walled smooth spermathecae. external head nuclear mushroom-like. Individual 184 μm long ducts tapeworms-like.

#### Comments about the *Idiophlebotomus* species

We caught a few specimens of *Idiophlebotomus* (*n* = 14). Our sampling could include two populations being identified as *Id. longiforceps* or a species closely related to this species. At this time, we prefer to identify all the specimens in the present study as *Idiophlebotomus sp.,* pending a revision of this genus with more specimens and molecular studies.

#### Comments about the genus *Chinius*

The genus *Chinius* had never been recorded in Vietnam. Our specimens were caught in caves. We identified *Ch. junlianensis* according the the short R2 vein of their wings [19] and to the length of the aedeagal and spermathecal ducts [6].

#### Comments about the genus *Grassomyia*

**Figure 4.**
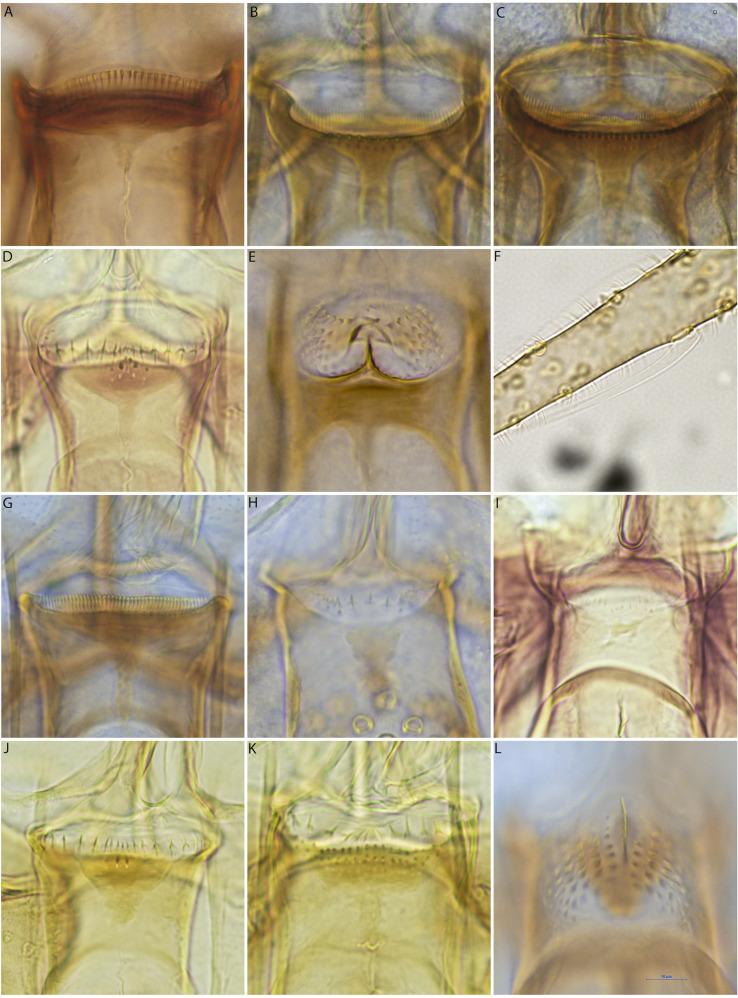
Microphotographs of cibaria of females of *Grassomyia indica* s. l*.* (A), the *Sergentomyia barraudi* group (B and C), *Se. khawi* (D), and the *Se. anodontis* group (E), ascoid of flagellomere 3 of *Se.* sp.3 with a retrograde spur, cibarium of the *Se. brevicaulis group* (G), cibarium of *Se. sylvatica* (H), cibarium of *Se. bailyi* (I), cibarium of *Se. hivernus* (J), cibarium of the *Se. perturbans* group (K), cibarium of *Idiophlebotomus* sp*.* (L), all at the same scale.

The taxonomic status of *Gr. indica* needs to be revised in the light of an integrative taxonomy of the whole genus, including all the species available and many populations from Africa, Madagascar and Asia. Pending this revision, we consider *Gr. indica* to be a valid species despite the synonymy proposed by Quate [33]. However, we observed several differences between the Indian specimens and those we studied in the present paper, i.e. the number of cibarial teeth ranged from 33 to 36 in the original description, whereas it ranged from 25 to 33 (Fig. 4A) in the present study. Without access to Indian specimens, we consider that our specimens belong to *Gr. indica* s. l.

#### Comments about the genus *Sergentomyia*

As described in the “Results” section, the *Se. barraudi* group should be further studied because of considerable heterogeneity in the morphological characters, such as the number and distribution of teeth on the cibarium. The cibarium in the original description made [41] from Indian specimens included 40 teeth and a forked anterior part of the sclerotized area. The specimens we examined showed a similar forked sclerotized area but 52 to 70 cibarial teeth (Figs. 4B, 4C), which appears quite different from the original description. Considering the different biogeographical subzones of the Indo-Malay area, it seems logical the populations from Vietnam are individualized from those from India, and possibly belong to different species. In our opinion, *Se. brevicaulis* can be split from the *Se. barraudi* group not taking into consideration the number of cibarial teeth, but taking into consideration the lack of a forked anterior part of the sclerotized area of the cibarium.

Many records of *Se. iyengari* have been made in Southeastern Asia. However, this species has been described from the most Southern part of India [43]. In our opinion, all the records of *Se. iyengari* in Southeastern Asia refer in fact to *Se. khawi* [37] (Fig. 4D).

Among the species historically considered junior synonyms of *Se. iyengari*, it clearly appears that the synonymization of *Se. hivernus* is wrong. *Sergentomyia hivernus* is now considered a valid species [30] which can be identified thanks to its wide spermatheca and to the low number of vertical teeth of the cibarium [33].

*Sergentomyia bailyi* has been described in India from specimens caught at different altitudes ranging from sea level to 1830 m above sea level [42]. Sinton explained that he observed considerable variability within his sampling, according to the altitude where the specimens orginated, suggesting a subspecies for specimens caught at high altitudes (*Se. bailyi campester*). Later, Raynal and Quate redescribed *Se. bailyi* from Southeast Asia [33, 34], in agreement with the original description but emphasizing variability in the length of the flagellomeres of the cibarial teeth, and the absence or presence of a sclerotized area of the cibarium. Consequently, we recorded *Se. bailyi* in Vietnam according to this supposed intraspecific variability pending a taxonomic revision.

*Sergentomyia sylvatica* was first described as *Se. sylvatica* from Vietnam [36] and the specimens collected in the present study are in agreement with the original description: a few teeth in the center of the cibarium and spermathecae not completely segmented.

The taxonomic status of *Se. perturbans* remains doubtful. Described from Indonesia [5], the cibarium of the female was described later from Chinese specimens [20, 29]; Lewis (1978) explained the confusion related to this species. Its status remains unclear. The capture of new specimens from different countries and their morphological study coupled to a molecular approach, will help us to better understand what *Se. perturbans* really is and to decide on the synonymizations made over the years. The specimens observed during the present study exhibit a few cibarial teeth and spermathecae in agreement with the redescription of Lewis [20], but they also exhibit some vertical teeth never recorded previously. Consequently, our records of *Se. perturbans* need to be approved by a systematic revision in the future.

In the present paper, we called *Sergentomyia* sp.2 (*Se*. sp2) the sandflies which are closely related to *Se. anodontis*. At the present time, we are not able to decide whether the specimens processed in the present study belong to *Se. anodontis* or to a new species. In the original description, the cibarium is described as “unarmed, but with spine-like projections from fold in membrane above sclerotized part and with median projection over which is inverted V-shaped bar” [32], and the drawing provided did not exhibit any lateral teeth. The specimens observed in Vietnam exhibit many lateral small teeth (Fig. 4E), differentiating this population from the original one. However, we do not know if this difference is of a specific or populational level. Moreover, the spermathecae of the specimens from Vietnam seem to be wider at their top than those described in the original description. We called *Se.* sp3 a *Sergentomyia* with a spur on the ascoid (Fig. 4F), which we think has not yet been described. We are waiting for more samples and molecular data to be able to describe it properly.

A heterogeneous pool of 83 specimens that we are unable to identify yet are here named undetermined species (und_sp*.*). We will need to carry out complementary analyses in order to be able to sort them out. New material and molecular data are needed as we think this pool may host new species to describe.

### Ecology and preference habitats according to the provinces

The distribution of sandflies, all species combined, is heterogeneous according to province in terms of species richness but also abundance and density. The largest numbers of sandflies were collected in Lang Son and Ninh Binh and the lowest number in Lao Cai (Tables 2 and S1). The differences between these provinces are probably due to the fact that Lang Son and Ninh Binh are rich in rounded rocky peaks, commonly called “sugar loaves”. It is worth noting that it is within these sugar loaves which shelter caves and crevices that 55.36% of sandflies were collected. All genera and species including the unknown *Se.* sp2 and *Se.* sp3 were reported in these specific environments. Previous studies carried out in Southeast Asia, as well as in Africa and South America, already reported that these environments are favorable to sandflies [3, 27, 46]. After caves and crevices, outdoor areas were the next preferred type of habitat, including gardens (Table 3), with 936 specimens collected in this environment, representing 36.21% of the sampling. Moreover, 16 sandfly specimens were collected in dog sheds, representing a density of 0.36, lower than the density found in caves (*D*_cave_ = 0.79) but higher than that found in outdoor areas (*D*_outdoor_ = 0.23) and other areas. In indoor environments and in bird/buffalo/pig sheds, the number of collected sandflies and their densities were very low. The statistical analyses showed that the distribution of species was not statistically different according to province but strongly different according to environment. The highest species richness values were found in caves and outdoors (SR = 15 including *Se*. sp2 and *Se*. sp3). In animal sheds and indoors, the species richness varied between five and nine, showing more anthropophilic behaviors or attraction for domestic animals for a few species such as *Ph. stantoni*. Ind fact, even though this species was found in all environments, it was the main species collected indoors (11/30 specimens, 36.67%) and in dog sheds (7/16 specimens, 43.75%). In caves, this species represented only 2.24% (32/1431 specimens). Further studies are necessary on this species in terms of feeding preferences since it was described as a cavernicolous species in Thailand and Malaysia [2, 31, 40]. As described in our previous paper focusing on Quang Ninh, two females out of nine specimens were found in the household of one of the leishmaniasis patients diagnosed in 2001 [10].

### *Leishmania* transmission risk to humans

Autochthonous visceral leishmaniasis was detected in the 2000s in Quang Ninh province and in 2018 in Quang Binh province [10, 48]. These cases raise the question of local *Leishmania* transmission in Vietnam. The isolate from one patient in Quang Ninh was identified by the Queensland International Institute, Brisbane, Australia as *Leishmania infantum* or *Le. donovani* (patient’s family, personal communication). It is worth noting that *Le. infantum* is a zoonotic parasite using dogs as its main reservoir and *Le. donovani* is an anthroponotic parasite. Thus, many questions remain unanswered.

As described above, in the six provinces under study, the majority of sandfly specimens were collected in cavernicolous environments and especially in sugar loaves, suggesting trophic preferences for cavernicolous wild animals, such as bats (Table 3). Sugar loaves are generally surrounded by houses either at the periphery of the towns or villages or in rural environments. This suggests possible contact between cavernicolous sandflies, humans and domestic animals. This is illustrated by the fact that all the species identified (including *Se.* sp2 and *Se.* sp3) were found in caves and outdoors, whereas species diversity was much lower indoors and in animal sheds (from 15 different species in caves to 5 in dog and pig sheds).

Combining all the data of the six provinces, out of 2585 specimens, only 46 specimens were found indoors or in dog sheds. The main species found in these environments was *Ph. stantoni* (18/46, 39.13%), with *Ph. stantoni* collected in the household of the patient diagnosed with leishmaniasis [10]. We cannot make any assumptions since until now, this species has been little studied and was never described as a vector of *Leishmania*. Nevertheless, two papers reported that human blood and non-identified *Trypanosoma* DNA were detected in *Ph. stantoni* in Thailand [30, 44].

Several studies strongly suggested that unexpected sandfly species could be able to transmit *Leishmania* [25, 37, 39]. Recently, the data published by Srisuton et al. [45] indicated that several species of sandflies might be potential vectors of *Leishmania* and *Trypanosoma* parasites in Southern Thailand. Thus, all the species and ecosystems need to be explored. In Vietnam, *Leishmania* could be transmitted by an unexpected sandfly species, and the transmission could occur in caves frequently visited by humans due to the presence of small temples. All these aspects underline the need to further study sandfly populations in Vietnam for vector identification and location of leishmaniasis transmission. In this context, the next steps are: to further study *Ph. stantoni*, to characterize all the Sp. specimens using detailed morphological and molecular studies, to explore the variability of the *Se. barraudi* group, to determine the feeding preferences of sandflies in the different ecosystems, to look for the parasite using PCR diagnosis, and to carry out field work in Quang Binh province, where the visceral leishmaniasis case was recently reported [48].

## Supplementary material

Supplementary material is available at https://www.parasite-journal.org/10.1051/parasite/2021080/olm *Table S1*. Total data collection.
